# Thoracic Spine Fracture-Dislocation After Minor Trauma in a Neurologically Intact Patient With Congenital Absence of Posterior Spinal Elements: A Case Report

**DOI:** 10.7759/cureus.101659

**Published:** 2026-01-16

**Authors:** Rawwaf Alfarsi, Mohamed Elkhalifa, Jaser Tashkandi, Mohammed Hadi

**Affiliations:** 1 Department of Orthopedic Surgery, King Saud Bin Abdulaziz University for Health Sciences College of Medicine, Jeddah, SAU; 2 Department of Orthopedic Surgery, King Abdulaziz Medical City/Ministry of National Guard-Health Affairs, Jeddah, SAU

**Keywords:** congenital anomaly, fracture-dislocation, neurologically intact, spinal instability, spontaneous decompression, thoracic spine

## Abstract

Thoracic spine fracture-dislocations are typically the product of high-energy injuries, and their neurological sequelae are usually devastating. For a patient to experience such an injury from a minor trauma and preserve all neurological function is rare and is often credited to the paradoxical decompression effect that follows a traumatic fracture of the posterior elements of the spine. This is a case of a 31-year-old female patient who suffered a severe thoracic spine fracture-dislocation from a low-energy fall from a manually operated “merry-go-round” in a kids' park. Despite the apparent instability, she was totally neurologically intact. Furthermore, MRI and CT were performed, which revealed a fracture-dislocation at the T4-T5 level and a previously undiagnosed congenital absence of the spinous processes and the posterior ligamentous complex at the level of T3-T6. The patient was successfully managed with open posterior reduction and instrumented fusion. Her recovery was complete and remains neurologically intact at long-term follow-up. In the context of severe spinal instability, this case highlights a unique pathophysiological mechanism for neurological sparing. The congenital absence of posterior elements of the thoracic spine created a biomechanically vulnerable segment that is susceptible to dislocation from such a minor trauma. Simultaneously, this also created an abnormally capacious spinal canal that protected the spinal cord from getting injured. This report highlights the importance of having a high index of suspicion for underlying congenital abnormalities when a significant spinal injury occurs from a low-energy mechanism.

## Introduction

Fracture-dislocations of the thoracic spine are considered one of the most severe injuries of the axial skeleton. They represent a catastrophic failure of all three of the spinal columns as described by Denis [1]. These injuries are usually the result of high-energy forces such as motor vehicle accidents and falls from significant heights [2,3]. The thoracic spine, stabilized by the rib cage, has an inherent rigidity. Moreover, the narrow dimensions of its spinal canal make such injury highly associated with the incidence of complete and permanent spinal cord injury [4,5].

Despite the poor prognosis, a small collection of case reports demonstrated a rare phenomenon of patients remaining neurologically intact despite the severe injuries [6-14]. The traumatic spontaneous decompression is the predominant mechanism proposed for the neurological intactness in such injuries [7,8]. The injury force, in these instances, also fractures the posterior elements of the spine (pedicles, facets, or lamina), causing the vertebral arch to become free-floating. This separation allows the vertebral body to dislocate as the posterior arch remains aligned. Subsequently, this leads to an effective enlargement of the spinal canal, which prevents the spinal cord from being injured [9,10,13].

This case report highlights a unique and novel etiological perspective to the existing literature. It describes a 31-year-old female patient who has an undiagnosed congenital absence of the posterior elements of the thoracic spine. The patient experienced a minor trauma that led to a complete fracture-dislocation of the thoracic spine. The preexisting congenital abnormality created a biomechanically weak spinal segment. As a result, it predisposed the patient to suffer a complete fracture-dislocation but paradoxically preserved neurological function.

## Case presentation

This is a 31-year-old female patient, medically free, who presented to the emergency department as a referral from a peripheral hospital. She sustained a trauma three days prior to the referral. While she was playing with her daughter in the playground, she fell off a manually operated merry-go-round on her head and rolled over a few times.

When the patient presented to our hospital, she was conscious, oriented, and vitally stable. She reported no history of loss of consciousness, bleeding, or vomiting when the trauma took place. Upon physical examination, the patient was lying in bed, conscious, alert, and oriented to time, place, and person. A complete neurological exam showed no deficit. The patient exhibited 5/5 motor strength in all extremities with intact sensation to light touch and pinprick. She had normal deep tendon reflexes and intact sphincter tone. She had a negative history of fever, chills, altered mental status, loss of consciousness, headache, seizures, focal neurological deficits, cough, upper respiratory tract infection (URTI) symptoms, abdominal pain, change in bowel habits, and urinary symptoms.

**Figure 1:**
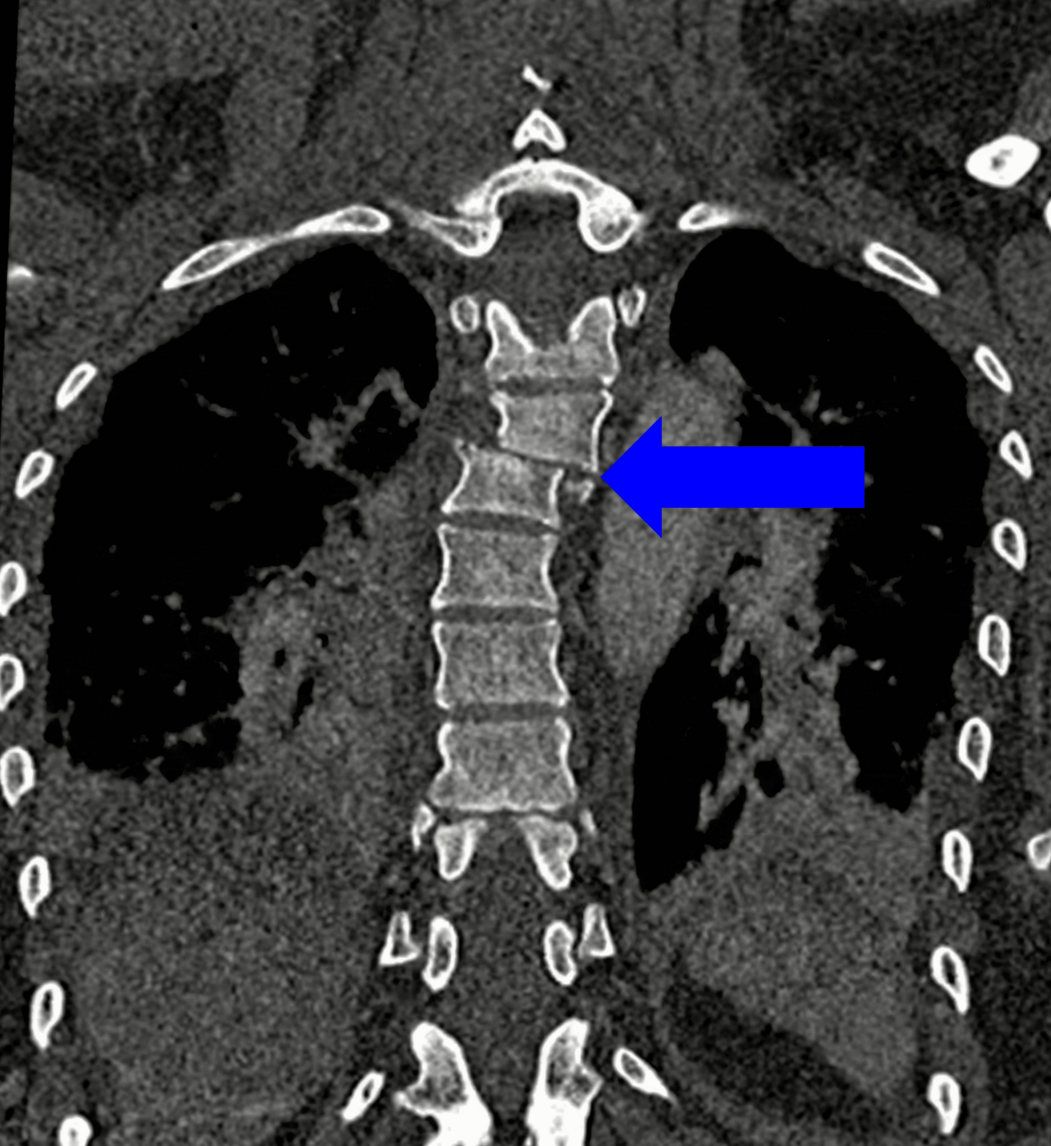
Coronal CT scan of the thoracic spine Coronal CT scan demonstrating the fracture-dislocation of the thoracic spine with significant lateral translation of the vertebral column.

**Figure 2:**
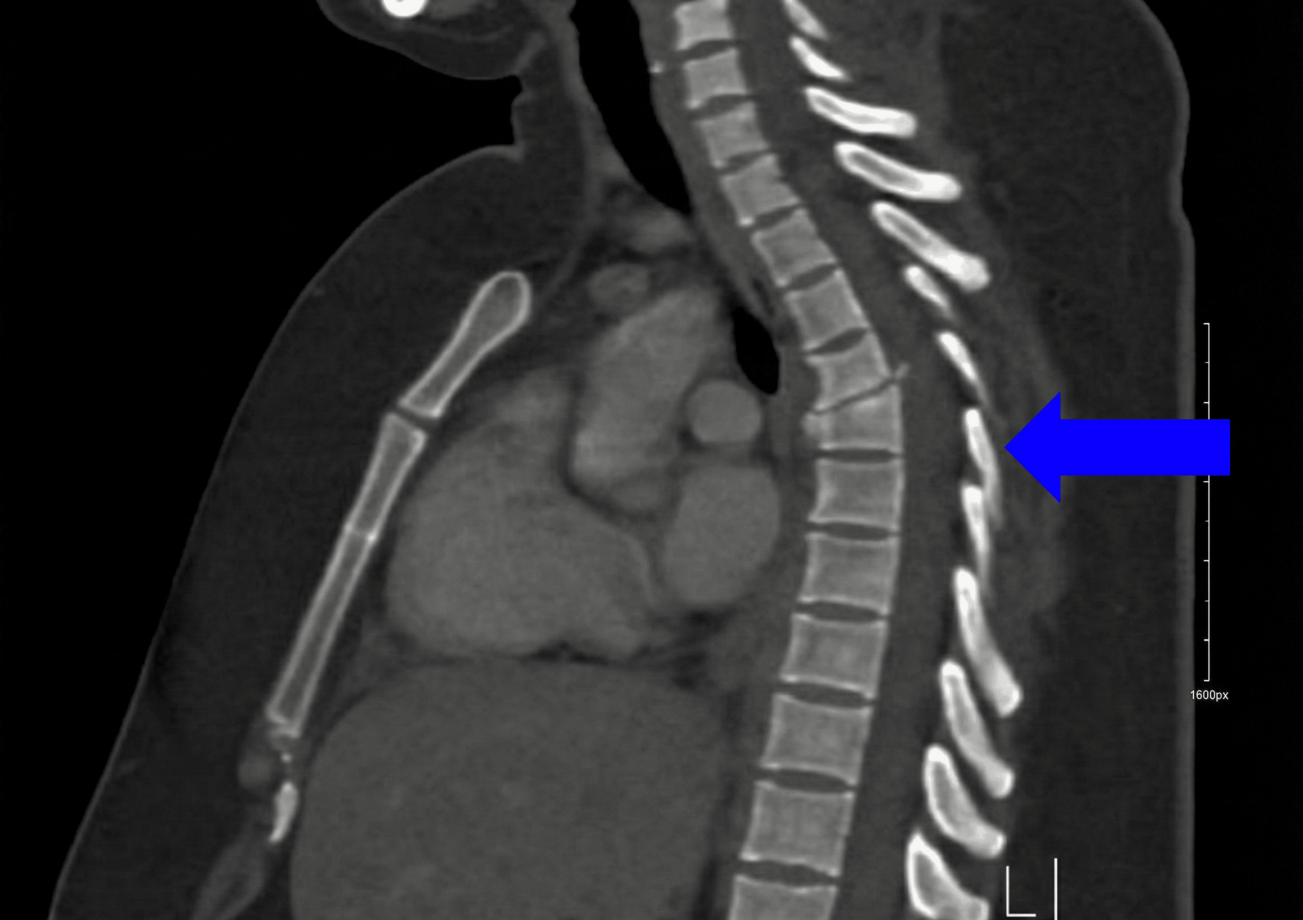
Sagittal CT scan of the thoracic spine Sagittal CT scan showing the congenital absence of the spinous processes.

**Figure 3:**
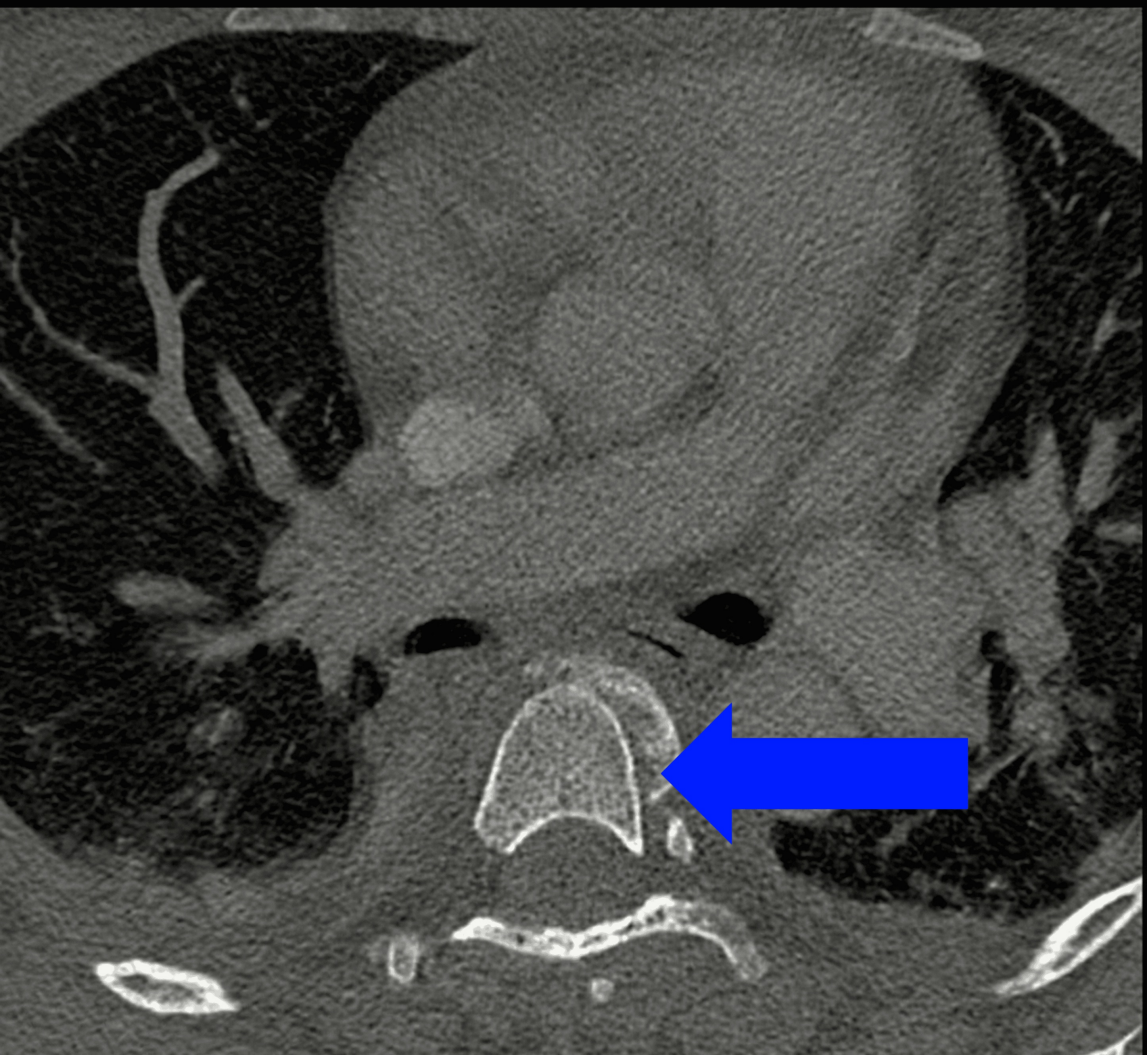
Axial CT scan of the thoracic spine Axial CT scan showing a fracture-dislocation at the level of T4-T5.

**Figure 4:**
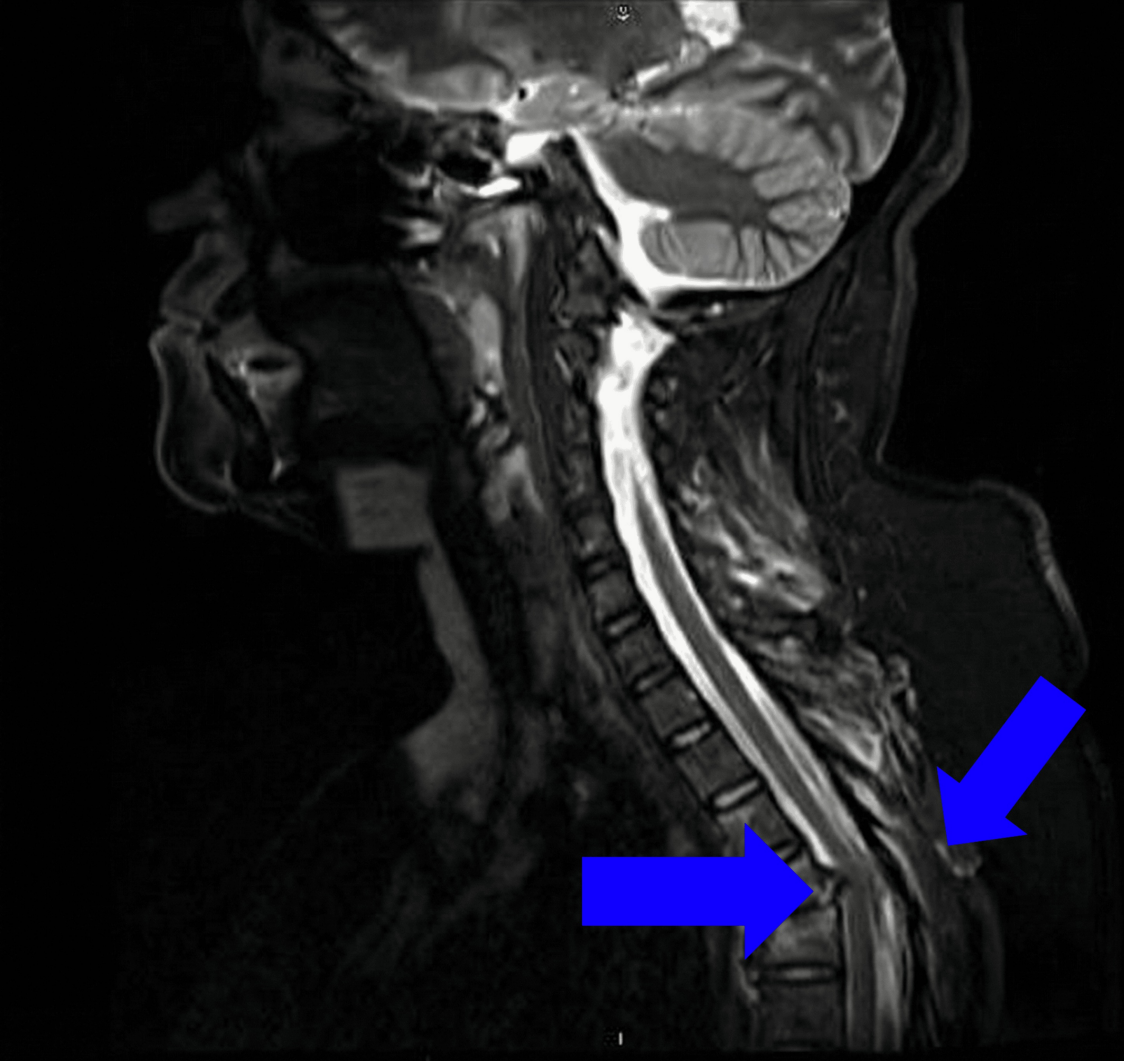
Sagittal MRI demonstrating the spinal cord MRI showed absence/agenesis of the posterior ligamentous complex, with no evidence of spinal cord compression, edema, or intramedullary hyperintensity.

Initial anteroposterior and lateral imaging revealed malalignment in the thoracic spine. Furthermore, a CT scan showed a fracture-dislocation at the level of T4-T5 (Figures 1-3). Moreover, the CT also demonstrated the congenital absence of the spinous processes of the affected vertebra (Figure 2). MRI showed absence/agenesis of the posterior ligamentous complex, with no evidence of spinal cord compression, edema, or intramedullary hyperintensity (Figure 4).

**Figure 5:**
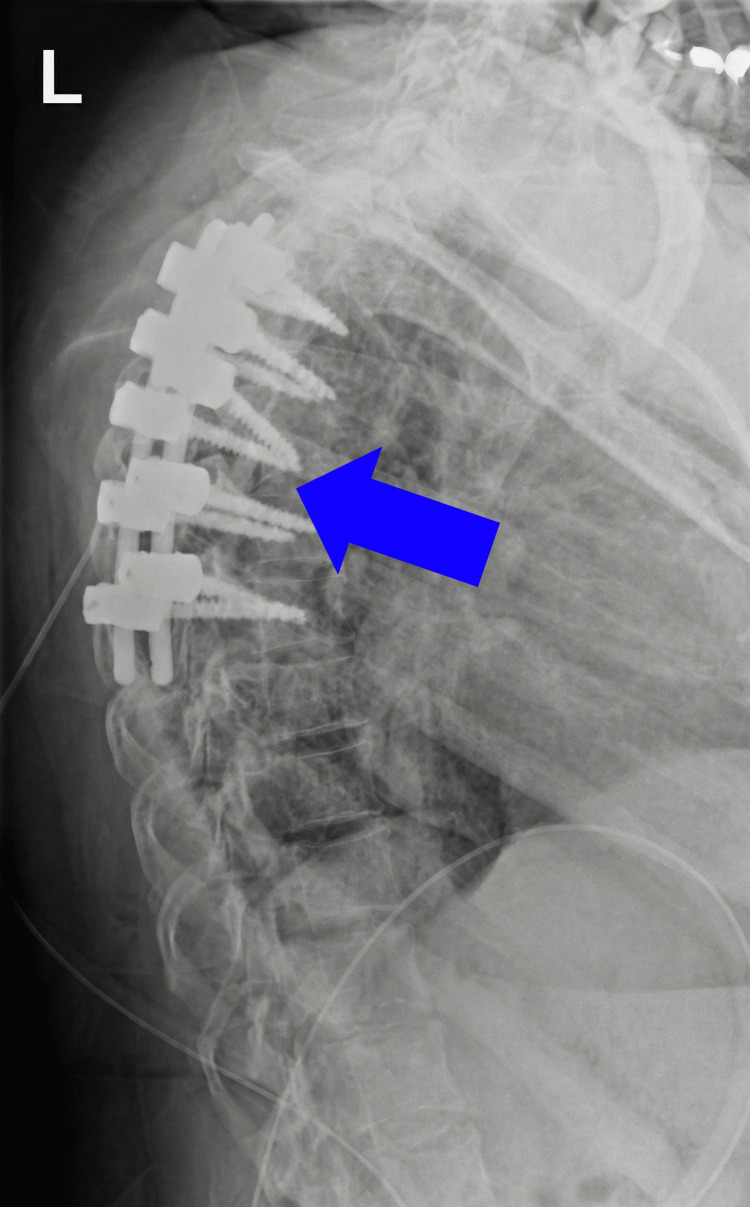
Postoperative lateral radiograph of the thoracic spine

**Figure 6:**
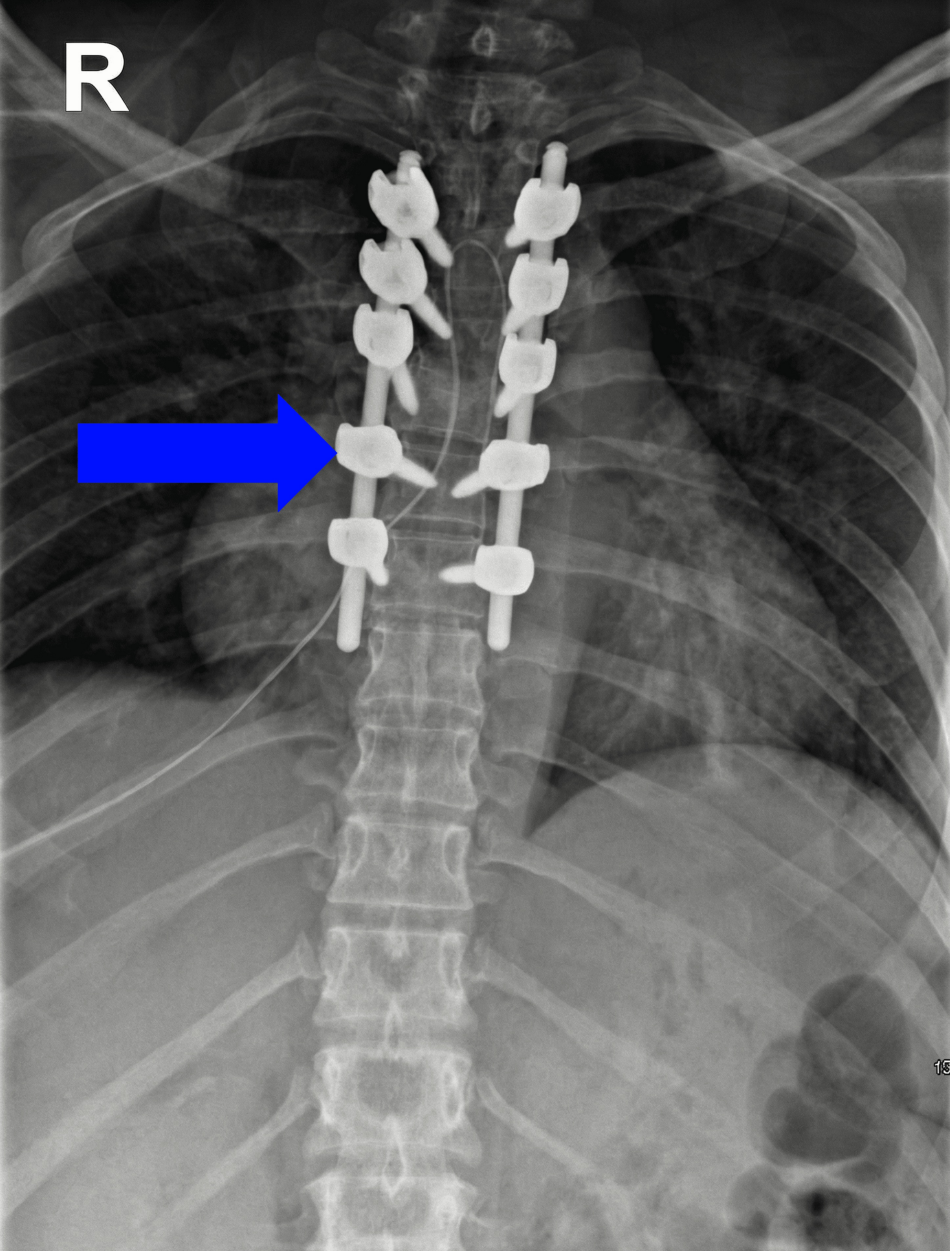
Postoperative anteroposterior (AP) radiograph of the thoracic spine

The patient underwent an urgent surgical stabilization, as mentioned above. Under general anesthesia, the patient was positioned prone on a Jackson table with proper padding of all the pressure points. Usual preparation and draping were performed. Cefazolin was administered preoperatively as prophylaxis. Skin marking was made with the help of the O-arm. A longitudinal skin incision was made, centered over the spine, exposing T3 down to T7. Subperiosteal exposure of the posterior elements of the spine was then carried out. There was an obvious dislocation at the right side facet T4-T5. Instrumentation was made with the help of the neuronavigation O-arm. There was an anomaly of the posterior elements of the spine, mainly the absence of spinous processes, supraspinous ligaments, and interspinous ligaments at multiple levels from T3 down to T6. Pedicle screws were inserted using the 5.5 mm Solera system. Satisfactory purchase of the bone was achieved, including the fracture site. T4/T5 facets were drilled and resected, resulting in a reduction of the fracture. A double-level laminectomy at T4 and T5 was performed, exposing the spinal cord with thorough decompression. Rod application and final tightening of all the screws were performed, with excellent reduction of the fracture (Figures 5, 6). Thorough washing and hemostasis were performed, followed by decortication of the posterior element of the spine for fusion. Local bone graft and allograft were applied to facilitate fusion. The skin was closed in layers, a drain was inserted, and a pressure dressing was applied. The patient tolerated the procedure very well and moved to recovery in good condition with intact neurological function of both lower limbs.

Postoperatively, she was vitally stable. She was conscious, alert, and oriented, doing well with no nausea, vomiting, or headache. Pain was controlled. She was tolerating orally and passing stool and urine. She started mobilizing the first day after surgery with no complications.

On follow-up at one year post-surgery, the patient was doing well. X-rays showed no abnormality.

## Discussion

In a complete thoracic spine fracture-dislocation, the complete preservation of neurological function is a rare event that challenges the conventional expectations of spinal trauma. The previous literature has primarily attributed this neurological intactness to one single mechanism, which is the traumatic disruption of the posterior vertebral arch. However, this case introduces a compelling alternative mechanism of preservation of complete neurological function. The preexisting congenital abnormality led to the same fortunate outcome but through different means.

The cases reported by Akay et al. [9], Phadnis et al. [11], Kumar et al. [13], and others report patients who experienced high-energy trauma without paralysis due to the spontaneous laminectomy that happened after the fracture of the pedicles or facets. This spontaneous laminectomy separated the vertebral body from the posterior elements, creating a significant increase in the functional diameter of the spinal canal at the moment of impact. Subsequently, this allowed the spinal cord to be spared from direct shearing or impingement injury [9,11,13].

Rahimizadeh et al. proposed another biomechanical model where a severe hyperflexion combined with shearing rotational force allows the spinal canal to remain aligned with facet joints becoming engaged and locked, causing dislocation, while the spinal cord acts as a pivot point, therefore saving it from transection [12].

This case shares the fundamental principle of a capacious spinal canal. However, it differs significantly in its origin. The canal was not widened due to the trauma; it was congenitally abnormal. The agenesis of the posterior elements of the thoracic spine is a rare condition, and its biomechanical consequence is inherent instability. The spine becomes highly susceptible to dislocation from low-energy trauma without the tension band of the posterior ligamentous complex and the bony support of the spinous processes. This explains the vulnerability to severe fracture-dislocation from such a trivial low-energy injury while paradoxically preserving a complete neurological intactness. Otherwise, the injury would have narrowed the canal and likely led to spinal cord transection during the dislocation.

The management of such injury, however, remains uncontroversial. As demonstrated consistently in the literature and in this case, regardless of the neurological status, the standard of care is urgent open reduction and long segment posterior instrumental fusion [3,10,13]. This approach of stabilization restores the physiological alignment of the spine, protects the spinal cord from secondary injury during patient mobilization, and provides an optimal environment for bony fusion, as seen in the excellent outcome in this patient.

## Conclusions

This case adds a significant and novel perspective to the literature on neurologically intact thoracic fracture-dislocations. It demonstrates that the congenital absence of the posterior elements of the spine can create a state of chronic underlying instability. As a result, this predisposes the patient to suffer a severe injury from a low-energy trauma. This case highlights that the absence of neurological deficit is not a reliable indicator of spinal stability and that healthcare practitioners should maintain a high index of suspicion for a potentially unstable spinal injury when the severity of radiographic findings is inconsistent with a low-energy trauma.
